# The Outcomes and Revision Rate of Total Hip Arthroplasty in a Single Tertiary Center: A Retrospective Study

**DOI:** 10.7759/cureus.27981

**Published:** 2022-08-13

**Authors:** Khalid A Alsheikh, Ali A Alhandi, Mutlaq S Almutlaq, Lina A Alhumaid, Naila Shaheen

**Affiliations:** 1 Department of Orthopedics, College of Medicine, King Saud bin Abdulaziz University for Health Sciences, Riyadh, SAU; 2 Department of Orthopedics, King Abdulaziz Medical City, Ministry of National Guard–Health Affairs, Riyadh, SAU; 3 College of Medicine, King Saud bin Abdulaziz University for Health Sciences, Riyadh, SAU; 4 College of Medicine, King Saud Bin Abdulaziz University for Health Sciences, Riyadh, SAU; 5 Department of Biostatistics and Bioinformatics, King Abdullah International Medical Research Center, Riyadh, SAU

**Keywords:** tertiary care centers, revision total joint arthroplasty, revision operation, revision joint replacement, total hip arthroplasty: tha

## Abstract

Introduction

Total hip arthroplasty (THA) is a commonly performed and successful orthopedic practice procedure. However, failure of arthroplasty may require revision THA and pose substantial clinical challenges for orthopedic surgeons. Therefore, this retrospective study aimed to estimate the revision rate of THA and its risk factors at a tertiary care hospital.

Methods

A retrospective cohort study was conducted in 2021 of patients who had undergone THA during 2016-2020 in a tertiary care hospital. All patients above 18 years old who had a THA were included in the study. The data was collected from patients' medical charts/electronic databases.

Results

A total of 148 THAs were included in this study. In total, 77 (52%) were females, and 71 (48%) were males. The average age of our patients was 49±17 years old, and the mean recorded BMI was 29.6. A total of 62% (n=92/148) of our participants were shown to have at least one comorbid disease, with hypertension being the most common comorbidity. Our findings show that half of the patients, 74 (50%), had a THA due to both primary and secondary osteoarthritis, 37 (25%) patients had avascular necrosis of the hip, and 25 (17%) were due to trauma. The most performed surgical approach was Kocher-Langenbeck (posterior) approach on 128 (86%), followed by the Hardinge (lateral) approach on 20 (13.51%). The most observed complication in the patients was postoperative pain in 35 (23.65%), followed by UTIs in 5 (3.38%). Of the 148 patients, nine (6.08%) had revision surgery. Regarding the revision rate, male patients were associated with a significantly higher rate of revision (P=<0.001), and older patients had a significantly increased risk of revision (P=0.026). Patients who developed complications, such as UTI, were associated with a higher revision rate (P=0.035). Also, a posterior approach (Kocher-Langenbeck) of the procedure was significantly linked to an increased risk of revision (P=0.014).

Conclusion

All in all, there are multiple associated factors with an increased incidence of revision THA. For example, male patients, older patients, complication development during the hospital stay, and posterior surgical approach were all associated with a significantly higher rate of revision.

## Introduction

Total hip arthroplasty (THA) is a commonly performed and successful orthopedic practice procedure [1,2]. It is a reconstructive procedure of the hip joint with an artificial prosthesis that has improved the management of many diseases that did not respond to conventional medical therapy. For this procedure, the two primary indications are severe pain and the limitation in activities of daily living that it causes. The most common condition for THA is osteoarthritis accounting for 70% of the cases [1]. Long-term studies of THA have proven the survival rate probability without sustaining a revision THA of 90% at ten years and 80% at 25 years [3]. Despite the excellent long-term outcomes and advances in surgical techniques, the number of revision procedures continues to increase [4,5]. Furthermore, failure of the arthroplasty and revision of THA remain substantial clinical challenges for orthopedic surgeons and their patients [4-6]. This study aimed to estimate the revision rate of THA and its complications at a tertiary care hospital.

## Materials and methods

Study design and population

This study is a retrospective cohort study conducted in 2021 of all patients who had undergone THA from 2016 to 2020 at King Abdulaziz Medical City (KAMC), Riyadh, Saudi Arabia. The study was approved (study number RC19/333/R) by the Institutional Review Board (IRB) of King Abdullah International Medical Research Center (KAIMRC), Riyadh, Saudi Arabia. The inclusion criteria for the study involved both males and females, age 18 and above, who had a THA procedure.

Data collection

The data was collected from patients' medical charts/electronic databases. The collected data included patients' demographic and clinical characteristics (age, BMI, gender, associated comorbidities, previous surgery), pre and post-operational length of stay in the hospital, surgical approach, complications (dislocation, fracture, aseptic loosening, prosthetic fracture, deep venous thrombosis, pulmonary embolism, UTI, hematoma, nerve injury, pain), revision THA, days between revision THA and primary THA, the surgical approach for revision, and two-year survival.

Statistical analysis

All collected data were processed and analyzed. Categorical variables such as gender were described in frequency and proportions, while the quantitative variables such as age and BMI were compared by analyzing means and SDs of corresponding data. Demographic and clinical characteristics were compared across the study groups using a t-test and Chi-squared test for continuous and categorical variables. All data are represented in percentage and mean values and were used for comparative analyses, with a corresponding 95% CI, and the statistical significance was considered at P ≤0.05. Age and length of stay were reported as mean, SD, and range. The covariates association with THA was assessed using Fisher's exact test. Results were reported as number/percentage and p-value. A p-value less than 0.05 was considered significant. Statistical analyses were carried out using SAS version 9.4 (SAS Institute, Cary, NC, USA).

## Results

A total of 148 THAs were included in this study. In total, 77 (52%) were females, and 71 (48%) were males. The average age of our patients was 49±17 years old, and the mean recorded BMI was 29.6. Also, 62% (n=92/148) of our participants were shown to have at least one comorbid disease, with hypertension being the most common comorbidity, followed by diabetes, dyslipidemia, and sickle cell disease with 30% (n=44/148), 18% (n=26/148), 18% (n=26/148), and 13% (n=19/148), respectively. In addition, 42 (28%) patients had previous surgery. Patients' demographics and clinical characteristics are presented in Table 1.

**Table 1:** Patient demographics.

Patient Demographics			
Gender:			
	Male N (%)	Female N (%)	
	71 (47.97%)	77 (52.03%)	
	Mean	Minimum	Maximum
Age	49.8	18	90
BMI*	29.6	16	46
	No (%)	Yes (%)	
Comorbidities:	56 (37.8%)	92 (62.2%)	
Hypertension	104 (70.3%)	44 (29.7%)	
Diabetes	122 (82.4%)	26 (17.6%)	
Previous stroke	145 (98%)	3 (2%)	
Dyslipidemia	122 (82.4%)	26 (17.6%)	
Sickle cell Disease	128 (87%)	19 (13%)	
Asthma	134 (90.5%)	14 (9.5%)	
Osteoarthritis	139 (94%)	9 (6%)	
Previous Surgery	106 (71.62%)	42 (28.38%)	

Table 2 shows that both the right and left sides had an equal number of 74 (50%) patients. The most performed surgical approach was Kocher-Langenbeck (posterior) approach on 128 (86%), followed by the Hardinge (lateral) approach on 20 (13.51%). The most observed complication in the patients was postoperative surgical pain in 35 (23.65%), followed by UTIs in five (3.38%). Furthermore, hematoma and nerve injury were the least seen in the patients, with one (0.68%) each.

**Table 2:** Causes, surgical approach, and length of stay. This table demonstrates the results of the causes, sides, and surgical approaches of each primary THA. In addition, it demonstrates the mean, minimal, and maximal length of stay pre and post-operatively.

Hospital Stay			
Causes:	N	(%)	
Osteoarthritis	74	(50%)	
Avascular Necrosis	37	(28%)	
Trauma	25	(17%)	
Rheumatoid Arthritis	3	(2%)	
Tumors	1	(0.6%)	
Side:			
Right	74	(50%)	
Left	74	(50%)	
Surgical Approach	N	(%)	
Kocher-Langenbeck (Posterior)	128	(86%)	
Hardinge (Lateral)	20	(14%)	
	Mean	Minimal	Maximal
Length of Stay	12	3	95
Length of Stay Post-operatively	8	3	46

**Figure 1:**
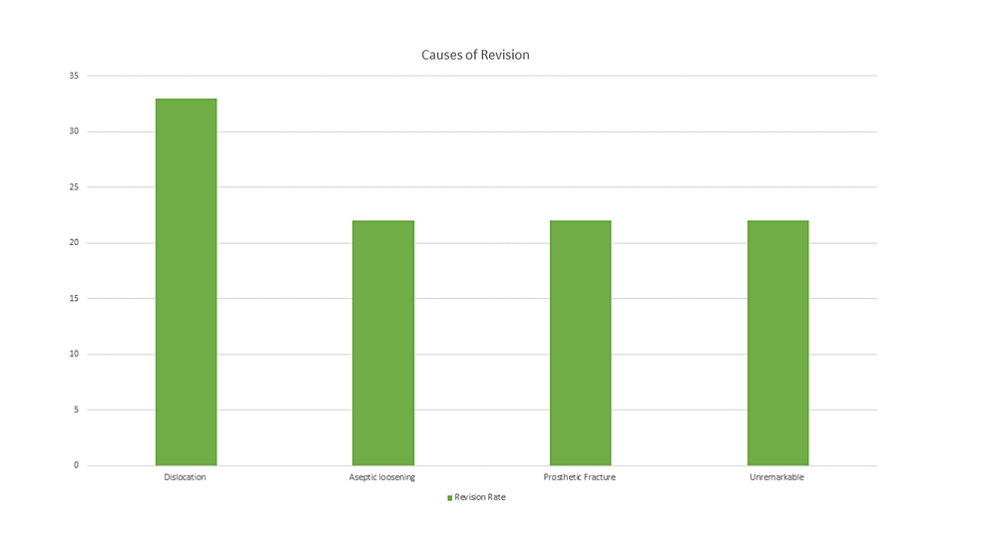
Causes of revision.

Out of the 148 patients, nine (6.08%) had revision surgery, as shown in Table 3. The revision rate is 6%, with 95% Wilson confidence limits 3.57-10.17. Three (2.03%) patients had a dislocation of the hip, two (1.35%) patients had the revision due to aseptic loosening, two patients had a periprosthetic fracture, and the final two patients' cause of revisions was unremarkable, as shown in Figure 1. The Kocher-Langenbeck (posterior) approach was used in six revision cases (66%), and the Hardinge approach was used in three of the cases (33%).

**Table 3:** Revision rate and the significant associations. This table shows the revision rate in all of the patients, and the surgical approach for each revision procedure. Moreover, this table reports the significant associations between each variable and the revision rate. THA*: Total hip arthroplasty.

Revision Rate				
	No (%)		Yes (%)	
Revision THA*	139 (94%)		9 (6%)	
Revision Surgical approach		N (%)		
Kocher-Langenbeck (Posterior)	6 (66%)			
Hardinge (Lateral)	3 (33%)			
Effect	Point Estimate		95% Wald Confidence Limits	95% Wald Confidence Limits
Age	1.054		1.006	1.104
Gender: Female vs Male	0.016		<0.001	0.499
Complications: No vs. Yes	0.188		0.035	1.020
Surgical Approach: Hardinge (lateral) vs. Kocher Langen beck (Posterior)	0.166		0.014	1.987
Length of Stay	1.098		1.031	1.169

Regarding the revision rate, male patients were associated with a significantly higher rate of revision (P=<0.001), and older patients had a significantly increased risk of revision (P=0.026). In addition, patients who developed complications, such as UTI, were associated with a higher revision rate (P=0.035). Also, a posterior approach (Kocher-Langenbeck) of the procedure was significantly linked to an increased risk of revision (P=0.014).

## Discussion

In our study, we observed factors associated with a higher revision rate following total hip replacement. However, it is often difficult to interpret individual risks that affect the outcome of the joint replacements, for it is influenced by the hospital environment, health care system, surgeons' expertise, and type of implant [7].

We found that male patients recorded a significantly higher rate of THA revision than female patients. In contrast, a study emphasized the risk for dislocation following THA and found that female patients had a higher risk of dislocation [8]. The findings in our study showed that older age had a higher chance of revision. Similarly, studies showed that age above 75 was at an increased risk of dislocation [9,10]. However, one study observed that the rate of revision-free survival for the older age group was higher than for the younger age group [3]. Another retrospective study done in the US discussed the relationship between revision rates of THA and age; there were 145 young patients (30 years old or younger) and 1,359 elderly patients (60 years old or older). The revision rates of the young population were 11% (16/145) and 3.83% (52/1359) for the elderly [11]. It may be due to the majority of our population being older with comorbidities and having a higher risk of falls.

The most observed cause for primary THA in our study was osteoarthritis, followed by avascular necrosis. However, there was no significant association with the revision rate. Likewise, the majority of patients undergoing THA in another study were due to osteoarthritis [2]. Another study showed that the primary indication for THA was predominantly osteoarthritis [12]. Furthermore, to our knowledge, no study found any significant association between the cause of THA and the revision rate. The question of which surgical approach for THA is the best has been heavily debated. In our study, we compared the surgical approaches for THA and their relation to revision rates. We found the posterior approach (Kocher-Langenbeck) to be linked to a higher revision rate. However, a study conducted in 2019 found the posterior approach to have a significantly lower overall complication rate than other surgical approaches [1]. Also, a study discussing THA revision due to dislocation concluded that the posterior approach increased the risk of revision for dislocation by 1.7 times [6,9]. Correspondingly, a posterior approach has been shown to be a major risk factor for dislocation due to reduced cup anteversion, which may be related to surgeon expertise [13].

Developing a complication following the THA procedure is not an indication for revision surgery; however, in our study, patients who had a complication postoperatively were associated with a higher revision rate. Severe surgical pain followed by UTIs were the most frequently observed complications. Furthermore, hematoma and nerve injury were the least observed. Unfortunately, no literature reviews were found regarding the association between postoperative complications and the revision rate. The most frequent causes for revision in our patients were dislocation followed by aseptic loosening and periprosthetic fracture. However, a French multicenter study found that aseptic loosening was the most common cause of revision, followed by periprosthetic fracture, and dislocation was the fifth cause [9]. Another study where the authors performed a complication-based analysis of total knee arthroplasty (TKA), THA, and total ankle arthroplasty (TAA) using worldwide arthroplasty registers reported the causes for revisions in THA were aseptic loosening (55.2%), dislocation (11.8 %), septic loosening (7.5%), and periprosthetic fractures (6%) [14]. Additionally, a study that consisted of 42,438 THAs focused on sepsis as the case of revision THA. The study concluded that out of the 42,238 THAs, 785 hips (2%) were revised for aseptic causes and 213 (0.5%) for septic causes [15].

Our study had several limitations. First, our study is a retrospective study, which means that some information could have been missed from patients' records. Second, surgical preparations, prosthetic types, and the lack of some secondary outcomes data were not documented, which may add to the factors related to the revision of the primary THA. Additionally, this is a single-center study, which limits the generalizability of the findings. Finally, one of the main limitations is being a single center with relatively small sample size.

## Conclusions

There are multiple associated factors with an increased incidence of revision THA. In our study, nine patients had undergone THA revision surgery. Male and older patients were associated with a significantly higher revision rate. Furthermore, patients who developed a complication during hospital stay had an increased risk of revision. Also, a posterior approach (Kocher-Langenbeck) is most likely associated with a higher revision rate. All in all, our study will contribute to medical knowledge and be a basis and a starting point for better patient care worldwide by being an indispensable element of aid in the prevention of developing complications. Therefore, future studies involving multicenter and a large cohort are required for further validations of our findings and appropriate precautions for modifiable risk factors.
